# Ultrastructural and immunohistochemical characteristics of telocytes in human scalp tissue

**DOI:** 10.1038/s41598-020-58628-w

**Published:** 2020-02-03

**Authors:** Li Wang, Li Xiao, Ruzhi Zhang, Huiling Jin, Haixia Shi

**Affiliations:** 1 Department of Dermatology, the Third Affiliated Hospital of Soochow University, 185 Juqian Road, Changzhou, 213003 Jiangsu Province People’s Republic of China; 2 Department of Dermatology, the First Affiliated Hospital of Bengbu Medical College, 287 Changhuai Road, Bengbu, 233030 Anhui Province People’s Republic of China

**Keywords:** Cell biology, Cells

## Abstract

This study was designed to characterize the location, morphology and ultrastructure of telocytes (TCs) in human scalp tissue. After obtaining approval for this study and informed consent from the patient, a scalp specimen was obtained. The distribution and morphology of TCs in human scalp tissue was assessed by immunohistochemical staining of CD34 and CD117/c-KIT, and the ultrastructure of TCs was investigated using transmission electron microscopy (TEM). Immunohistochemical staining of CD34 revealed that TCs were located in the connective tissue of human scalp, and were concentrated around hair follicles (HFs), blood vessels, sweat glands, sebaceous glands and adipose lobules. Immunohistochemical staining of CD117 revealed that TCs were mainly located in the dermis of human scalp, surrounding the HFs and sweat glands. Under TEM, TCs were seen and confirmed by their special morphological features. These cells were spindle-shaped, had small cell bodies and long thin processes, and surrounded stem cell clusters in the bulge region of HFs. These results demonstrate that TCs in human scalp were positive for CD34 and CD117, and their strategic positioning surrounding stem cells suggests their possible involvement in local regeneration, remodeling and homeostasis of the skin.

## Introduction

Telocytes (TCs) are a distinct type of interstitial cell that was first identified by Popescu’s group in 2005 and was officially named in 20101. TCs exist in various organs and tissues and are characterized by small cell bodies and one to five extremely long (ten to one hundred μm) and thin (less than 0.2 μm) processes called telopodes (Tps)^1^. Tps consist of alternations of podomers (80–100 nm) and podoms (~300 nm)^2^, the latter of which may contain mitochondria, caveolae (Ca2+ uptake/release units) and endoplasmic reticulum (ER)^1,3,4^. In addition to the unique features of Tps (with a very long and very slender, moniliform appearance), more and more evidence has emerged indicating that TCs differ from intersititial cells of Cajal and other types of interstitial cells (e.g. fibroblasts, fibrocytes, fibroblast-like cells and mesenchymal cells) based on their expression of cell surface antigens and microRNA profiles, and their gene expression and proteomic signatures^1,5,6^. The shape of TCs depends upon the number of the Tps arising directly from their cell body: pyriform for one Tp, spindle for two Tps, triangular for three Tps and stellate for four or more Tps^7–9^. In recent years, researchers have used focused ion beam scanning electron microscopy to investigate the 3D imaging of TCs in human dermis, and have revealed the existence of Tps with various conformations: (i) long, flattened irregular veils (ribbon-like segments) with knobs, corresponding to podoms, and (ii) tubular structures (podomers) with uneven calibre because of irregular dilations (knobs) – the podoms^10^. So far, TCs have been detected in many tissues and organs, including the heart^11,12^, lung^13,14^, skeletal muscle^15^, skin^16,17^, eye^18,19^, liver^20^, uterus^21^ and urinary system^22,23^.

TCs occupy strategic positions in tissues as they are located in the close vicinity of stem cell niches, vascular structures and nerves^24,25^. Furthermore, TCs develop close relationships with other types of cells, including mast cells, fat cells, plasma cells, basophils, lymphocytes and connective fiber bundles (collagen and elastic fibers)^26,27^. TCs can build a 3D network throughout the entire stromal space, particularly by the branching of their long and bead-like Tps^28,29^. TCs can communicate with each other and with other cell types by homocellular and heterocellular junctions, respectively^29^. In addition, increasing evidence has indicated that TCs play significant roles in regulating tissue morphogenesis and homeostasis, the guidance of tissue-resident stem/progenitor cell self-renewal and differentiation, as well as in various pathologies^26,30–34^.

There have been many studies about the existence, distribution, morphology, ultrastructure and the potential roles of TCs in different organs of the human body and in some animals in healthy and in diseased states. However, studies focused on the characteristics of TCs in human scalp are relatively rare. Therefore, this study was designed to investigate the existence, distribution, morphological characteristics and ultrastructure of TCs in the human scalp, using immunohistochemistry and transmission electron microscopy (TEM).

## Materials and Methods

### Ethical statement

This study was approved by the Ethics Committee of the Third Affiliated Hospital of Soochow University and conformed with the basic principles of the Declaration of Helsinki. Written informed consent was obtained from the patient.

### Treatment of scalp tissue

A small piece of scalp tissue (2 cm × 3 cm) was obtained from a young man (21–35 years) undergoing a routine plastic surgery in the Department of Dermatology. The sample was kept under sterile conditions and was divided into small pieces (1 cm × 1 cm), which were fixed in 4% buffered formalin, then were dehydrated in a graded ethanol series and xylene, and finally were embedded in paraffin. Serial sections of 5 μm thick were obtained using a rotary microtome (Leica RM 2135, Nussloch, Germany). The sections were deparaffinized and hydrated, then used for immunohistochemical analysis.

### Immunohistochemical staining

An indirect immunoperoxidase method was used in the study. After having been subjected to routine immunohistochemical preparation, the sections were incubated overnight at 4 °C with primary antibodies specific for CD34 (1:100; cat. no. ab81289, Abcam, Cambridge, UK) and CD117 (1:50; cat. no. ab5505, Abcam, Cambridge, UK) diluted in 1% bovine serum albumin in PBS (cat. no. SH30256.01, HyClone, Logan, UT, USA). The secondary antibody used was Goat Anti-Rabbit IgG H&L (HRP) (1:500; cat. no. ab96899, Abcam, Cambridge, UK). The sections were finally counterstained with haematoxylin, then washed and mounted in an aqueous mounting medium. Double stain of CD34 and CD117 was performed in the same way. Negative controls were processed in the same manner except for replacing the primary antibody with PBS. Sections were examined using an IX73 fluorescence microscope (Olympus, Tokyo, Japan) and images were obtained and analyzed.

### Transmission electron microscopy (TEM)

The scalp specimen was trimmed into a small piece (approximately 1 mm^3^) and was fixed in 2.5% glutaraldehyde (buffered to pH 7.3 with sodium cacodylate) for 4 h at 4 °C, and then was washed with 0.1 mol/L cacodylate buffer. The specimen was post-fixed in 1% osmium tetroxide in 0.1 mol/L cacodylate buffer at a pH of 7.3 for 1 h. Sequentially, the specimen was dehydrated using ethanol at concentrations of 30%, 50%, 70%, 80%, 90% and 100%, each phase for 10 min. This was followed by overnight impregnation with propylene and epoxyresin (Epon 812). Ultrathin sections (70 nm thick) were obtained using a Leica Ultracut UCT ultramicrotome (EM UC6, Leica, Wetzlar, Germany). These sections were placed onto a copper grid and stained with lead and uranyl solutions, after which they were observed and photographed using a TEM (JEM-2100, JEOL, Tokyo, Japan).

## Results

### Immunohistochemical staining

**Figure 1:**
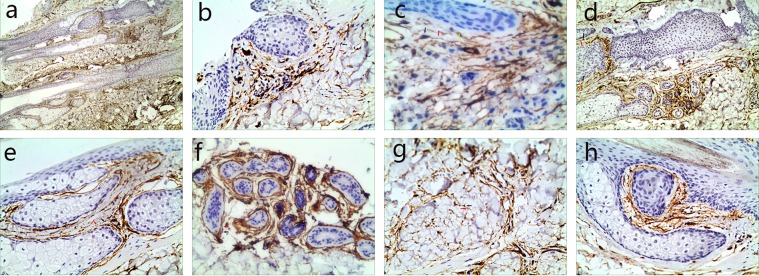
Human scalp tissue stained immunohistochemically for CD34. (**a**) CD34^+^ cells (TCs) (black arrow) were in the deep dermis and subcutaneous tissue (40×). (**b**) A few TCs body (black arrows) with their Tps (red arrows) extended parallel to the adjacent epidermis (100×). (**c**) The spatial relationship of TCs body (black arrows) with their Tps (red arrows) and a blood vessel (yellow arrow) (100×). (**d**–**g**) TCs body (black arrows) and their Tps (red arrows) surrounded deep segments of HFs (**d**, 100×), sebaceous glands (**e**, 200×), secretory or ductal parts of the sweat glands (**f**, 200×) and intervals of adipose lobules (**g**, 200×). (**h**) TCs body (black arrows) and their Tps (red arrows) bordering the bulge and sub-bulge areas of HFs (200×).

**Figure 2:**
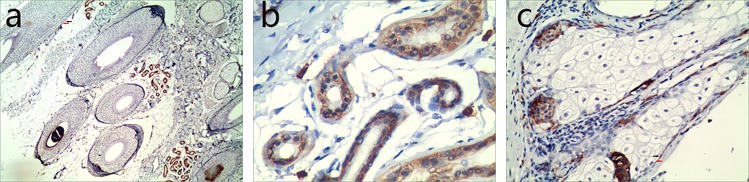
Human scalp tissue stained immunohistochemically for CD117. CD117+ cells (TCs body) (black arrows) with their Tps (red arrows) were observed surrounding HFs (**a**, 100×), sweat glands (**b**, 200×) and sebaceous glands (**c**, 200×).

**Figure 3:**
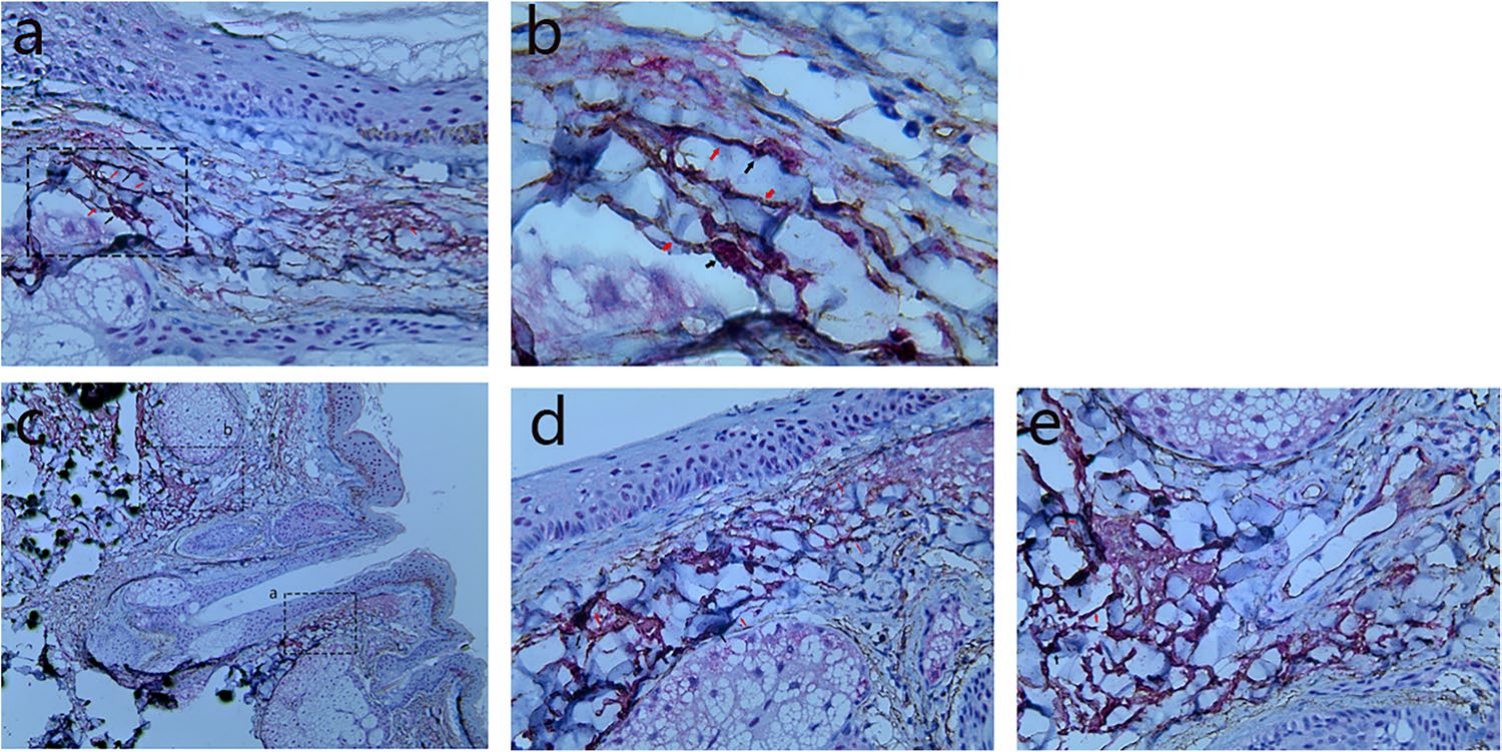
Human scalp tissue stained immunohistochemically for CD117 and CD34. CD117+ cells (TCs body) (black arrows) with their Tps (red arrows) were observed in the reticular dermis, subcutaneous tissue of normal scalp (**a**, 200×; **b**, 400×), and around the middle of HFs (**c**, 100×; **d**, 200×; **e**, 200×). Figure (**b**) is an enlargement of the rectangular frame of Figure (**a**). Figure (**d**) is an enlargement of the rectangular frame a of Figure (**c**). Figure (**e**) is an enlargement of the rectangular frame b of Figure (**c**).

Immunohistochemical staining revealed that CD34-positive (CD34+) cells were predominantly distributed in the reticular dermis and subcutaneous tissue of normal scalp, although a few were spotted in the papillary dermis (Fig. 1a,b). CD34+ cells were frequently observed in the perivascular connective tissue, accompanying small and intermediate blood vessels, and possessed characteristic cellular processes that extended parallel to the adjacent epidermis (Fig. 1b,c). The number of visible Two or three Tps were usually observed for each CD34+ cell. In the deep dermis and subcutaneous tissue, CD34+ cells surrounded deep segments of HFs (Fig. 1d), sebaceous glands (Fig. 1e), arrector pili muscles, secretory or ductal parts of sweat glands (Fig. 1f) and intervals of adipose lobules (Fig. 1g). Surrounding skin adnexa, CD34+ cells formed intermingled networks and created two to three incomplete concentric sheaths. It is worth noting that in the bulge and sub-bulge areas of HFs, where stem cells were numerous, CD34+ cells were more abundant and they were not only connected to each other, but also seemingly bordered clusters of stem cells (Fig. 1h). On the other hand, CD117-positive (CD117+) cells with Tps were observed surrounding HFs (Fig. 2a), sweat glands (Fig. 2b) and sebaceous glands (Fig. 2c). Sections used as controls with no antibody showed there were no false positive reactions. Double staining revealed that both CD34 and CD117 -positive cells were predominantly distributed in the reticular dermis, subcutaneous tissue of normal scalp (Fig. 3a,b), and around the middle of HFs (Fig. 3c–e).

### Analysis by TEM

**Figure 4:**
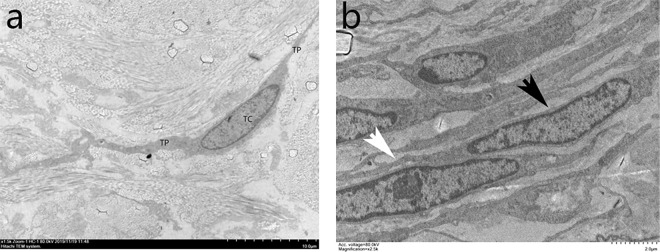
Ultrastructural analysis of TCs in the dermis of human scalp. TCs presented two or three extremely long and thin Tps (white arrows), which were close to the basal layer of the epidermis (**a**, 1500×) and ran parallel to it (**b**, 2,500×).

TCs in the dermis of normal scalp had two or three extremely long and thin processes, which ran parallel and close to the basal layer of the epidermis (Fig. 4a,b). These cells wrapped around blood vessels, sweat glands, sebaceous glands and HFs. Typically, two or three layers of TCs formed an incomplete sheath that wrapped around skin adnexa.

**Figure 5:**
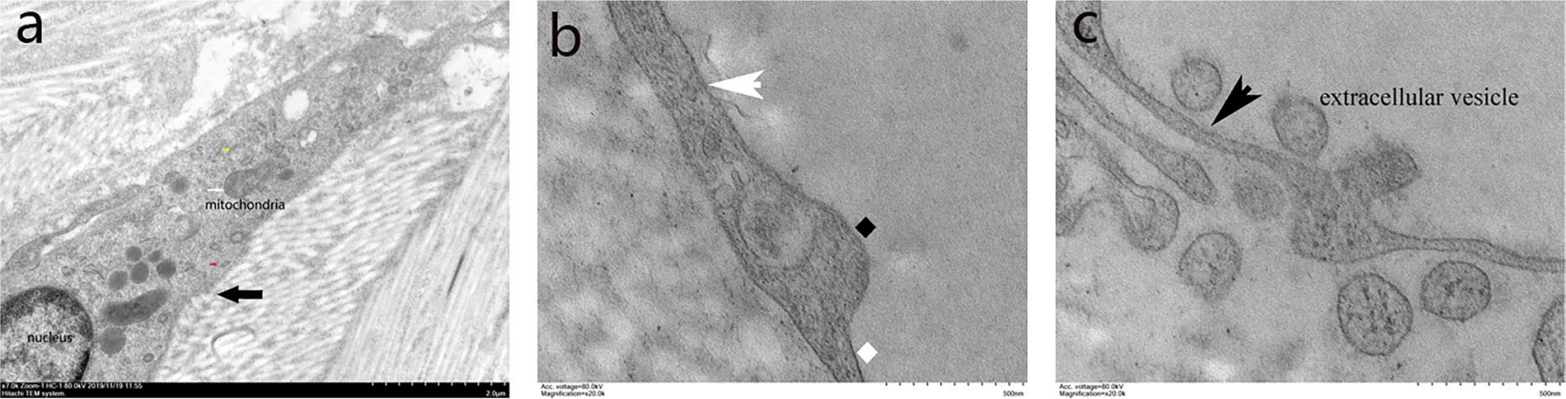
The fine structure of TCs (black arrow). TC mostly occupied by a nucleus and encircled by a small quantity of cytoplasm. The cytoplasm contained mitochondria (red arrow), rough (yellow arrow) and smooth (red arrow) endoplasmic reticulum (**a**, 7,000×). (**b**) Narrow segments (podomer, white rhombus) on a Tp (white arrow) alternating with dilations (podoms, black rhombus) (20,000×). (**c**) Extracellular vesicles released by a TC (20,000×).

TCs had small oval bodies, which were mostly occupied by an ovoidal euchromatic nucleus and encircled by a small quantity of cytoplasm. The cytoplasm contained mitochondria, rough and smooth ER (Fig. 5a). TCs presented with two or three extremely long and thin processes called Tps, which were often collagen-embedded or lining elastic fibres. There were narrow segments (podomers) on the TPs alternating with dilations (podoms) (Fig. 5b), which had released a few extracellular vesicles (EVs) that play an important role in cellular communications in the skin (Fig. 5c).

## Discussion

TCs have been regarded as a new type of cell. The immunohistochemical staining profile of TCs may be distinct between organs and may even be distinct within the same location1. Studies have revealed that TCs are positive for CD117/c-KIT, CD34 (hematopoietic stem cell marker, membrane protein), vimentin, caveolin-1, platelet-derived growth factor receptor α, and so on^35^. While specific immunohistochemical markers of TCs have not yet been identified, TCs in human dermis can be divided into two groups: one that expresses CD117 and another that expresses CD344. In our study, most CD34+ cells were detected in the reticular layer of the dermis and around vascular structures, sweat glands, sebaceous glands, HFs and fat lobules. CD34+ cells formed a 3D stromal network through homocellular and heterocellular junctions that is critical to maintaining the normal structure of the dermis. Only a few CD34+ cells were found in the papillary layer, results that are consistent with a previous study^36^. One slight difference noted in our study was that CD117+ cells appeared to be more pronounced around sweat glands in the scalp tissue.

Scalp tissue was chosen for our research since our purpose was to observe the spatial relationships between the locations of TCs and HFs. As far as we know, multiple stem cell niches have been observed in the skin, including the basal layer of the interfollicular epidermis, the bulge of HFs, the base of sebaceous gland dermal papillae and perivascular spaces^8^. Our immunohistochemical results showed that CD34+ cells are mainly located at the excretory ducts of eccrine sweat glands, blood vessels and the infundibulum of HFs, which suggests that TCs may reside in stem cell niches, and might participate in “nursing” the stem cells. The bulge of the HF is the best characterized stem cell compartment in the skin, located between the opening duct of sebaceous glands and the attachment point of arrector pili muscles. Based on the ‘bulge activation hypothesis’, bulge stem cells will proliferate and differentiate when they receive the necessary signals^4,37^. It is known that stem cells are not connected to each other by classical junctions, so the question arises: what is the carrier of those signals?

At the ultrastructural level, TCs present as small cell bodies with several long and thin Tps whose lengths vary from ten to one hundred μm. The Tps extend with alternations of podomeres and podoms, and they surround stem cells to form niches. According to those locations and morphological characteristics, increasing evidence has revealed that TCs may be key cellular elements involved in regulating tissue morphogenesis and homeostasis, the guidance of tissue-resident stem/progenitor cell self-renewal and diferentiation, as well as in various pathologies^26,30–34^.

TCs can establish two distinct kinds of connections: long distance connections via Tps and short distance connections via EVs. TCs can establish homocellular contacts with each other and heterocellular contacts with different cell types and non-cellular elements. The homocellular contacts might be significant in exchanging, relaying and passing information between TCs, while the heterocellular contacts might help with distant signaling, which is essential in tissue renewal. Point contacts and planar contacts were observed between TCs and putative stem cells.

In addition to these atypical heterocellular junctions, TCs can contact other cells (including stem cells) via their shed vesicles. Previous studies have reported that TCs can release three kind of EVs: exosomes, ectosomes and multivesicular cargos^33^. Those vesicles may act as intercellular shuttles to deliver biological signals. Usually, EVs are involved in intercellular signaling in normal and in diseased states by sending important macromolecules (e.g. RNAs, proteins or microRNAs) to adjacent cells. By these means, TCs integrate various types of information, from nerve cells, vascular cells, stem cells or immune system cells. In the future, it will be very important to determine the possible functions of TCs in stimulating resident stem cells to proliferate and differentiate for use in skin engineering and regenerative medicine. Recently, in consideration of the strategic location of TCs, some studies have been performed to account for their functions in pathologies and in regenerative medicine, including skin pathologies such as systemic sclerosis^38^, psoriasis^39^, basal cell carcinoma and squamous cell carcinoma^40^. Given the spatial relationship between stem cells and TCs in HFs, it may be promising to investigate variations of biological properties of melanocyte stem cells after co-culture with TCs since that may modulate their expression of the c-KIT receptor (CD117), their proliferation and migration and/or their ability to produce melanin. Exploring the mechanism of the effects of TCs on melanocyte stem cells may provide new approaches for the therapy of human pigmentary diseases.

In summary, TCs in human scalp express both CD117 and CD34, and they form 3D interstitial networks contacting the surrounding cells/structures by their Tps and/or by EVs. The spatial relationship between TCs and stem cells in the bulge of the outer root sheath in HFs may imply an important role for TCs in regulating hair and skin regeneration. The number, distribution and normal ultrastructure of TCs varied in pathological conditions of the skin.

## Conclusions

Thus, our results reveal that TCs are a distinct cellular entity in human scalp. Our study lays a foundation for studies on the roles of TCs in modulating the biological properties of melanocyte stem cells and even in some skin diseases, such as alopecia. To fully understand the roles of TCs in the skin, further study is warranted using dynamic *in vitro* and *in vivo* methods. Uncovering the potential functions of TCs in the skin to regulate the growth cycle of HFs may shed new light on their possible applications in hair regeneration.

## Data Availability

All relevant data are within the paper.
